# The effect of emotion regulation on risk-taking and decision-related activity in prefrontal cortex

**DOI:** 10.1093/scan/nsz078

**Published:** 2019-10-31

**Authors:** Carmen Morawetz, Peter N C Mohr, Hauke R Heekeren, Stefan Bode

**Affiliations:** 1 Department of Education and Psychology, Freie Universität Berlin, Berlin, Germany; 2 Center for Cognitive Neuroscience Berlin, Freie Universität Berlin, Berlin, Germany; 3 Center for Medical Physics and Biomedical Engineering, Medical University of Vienna, Vienna, Austria; 4 School of Business & Economics, Freie Universität Berlin, Berlin, Germany; 5 Markets and Choice, WZB Berlin Social Science Center, Berlin, Germany; 6 Melbourne School of Psychological Sciences, The University of Melbourne, Melbourne, Australia; 7 Department of Psychology, University of Cologne, Cologne, Germany

**Keywords:** fMRI, neuroimaging, emotion regulation, reappraisal, valuation, cognitive control, decision-making, risk-taking

## Abstract

Emotion regulation impacts the expected emotional responses to the outcomes of risky decisions via activation of cognitive control strategies. However, whether the regulation of emotional responses to preceding, incidental stimuli also impacts risk-taking in subsequent decisions is still poorly understood. In this study, we investigated the interplay between the regulation of incidentally induced emotional responses and subsequent choice behavior using a risky decision-making task in two independent samples (behavioral and functional magnetic resonance imaging experiment). We found that overall, emotion regulation was followed by less risky decisions, which was further reflected in an increase in activation in brain regions in dorsolateral and ventrolateral prefrontal cortex and cingulate cortex. These findings suggest that altering incidental emotions using reappraisal strategies impacts on subsequent risk-taking in decision-making.

## Introduction

Trading off risk and reward, for example when working as a stockbroker or making financial decisions as a company manager, has long been thought to be driven by a purely cognitive and logical assessment of risk (Westbrook and Braver, 2015). However, contrary to financial economic accounts of traders as rational utility maximizers, evidence is building that internal factors such as emotions (Lerner *et al.,* 2004; Phelps *et al.,* 2014) and the ability to regulate emotions (Lerner *et al.,* 2015; Heilman and Houser, 2016) have important effects on their financial decisions.

Thus, effective emotion regulation might be a relevant facet of trader expertise as it diminishes susceptibility to cognitive and psychological biases (Sokol-Hessner *et al.,* 2009; Heilman *et al.,* 2010; Fenton-O’Creevy *et al.,* 2012). Most studies investigating the interplay of emotion regulation and decision-making focused on the regulation of emotions that participants attach to possible outcomes that they anticipate as a consequence of making decisions (Delgado *et al.,* 2008; Grecucci *et al.,* 2013; Gu *et al.,* 2013; Hutcherson *et al.*, 2012; Martin and Delgado, 2011; Sokol-Hessner *et al.*, 2013; Sokol-Hessner *et al.,* 2009; Staudinger *et al.*, 2011; Staudinger *et al.,* 2009; van’t Wout *et al.,* 2010; van’t Wout *et al.*, 2013; Yang *et al.,* 2013). For instance, before making a risky decision, decision-makers can actively attempt to change the way they will perceive potential choice outcomes to minimize the emotional impact on decision-making. In this case, such emotions directly related to choice outcomes could either occur during decision-making (current emotions) or after decision-making (expected emotions) (Loewenstein *et al.,* 2001; Knutson and Greer, 2008; Schlösser *et al.,* 2013; Lerner *et al.,* 2015).

Only few studies have investigated the effect of regulation of experimentally induced emotion on risky financial decisions. One prominent way to minimize the effects of expected emotions on decision-making is reappraisal, the reframing of the meaning of an emotional stimulus to alter its emotional impact (Gross and Thompson, 2007). Engaging in reappraisal of the decision-situation has been shown to effectively reduce loss aversion (Sokol-Hessner *et al.,* 2009) and increase risk-taking (Braunstein *et al.*, 2014). Other studies revealed that greater habitual use of cognitive reappraisal was associated with a performance advantage in financial decision-making (Fenton-O’Creevy *et al.,* 2011) and with increased risk-taking, accompanied by decreased sensitivity to changes in risk-related probabilities and task configurations (Panno *et al.,* 2013). In contrast, the implementation of imagery-based emotion regulation (i.e. imagining relaxing scenes) before financial decision-making was associated with reduced risk-taking (Martin and Delgado, 2011).

However, there is also a rich literature showing the influence of incidental emotional stimuli on decision-making in general (Lerner and Keltner, 2001; Loewenstein and Lerner, 2003; Lerner *et al.,* 2004; Winkielman *et al.,* 2005; Han *et al.,* 2007; Pham, 2007; Seo and Barrett, 2007; Schulreich *et al.,* 2014). Similarly, several studies highlighted the carry-over effects of emotionally loaded stimuli, such as erotic images, brand logos and other highly valenced stimuli, on subsequent financial decisions (e.g. Murawski *et al.,* 2012; Wilson and Daly, 2004). Surprisingly, while these stimuli are designed to elicit strong emotional responses, the effect of emotion regulation for such incidental stimuli on subsequent decisions has not received much attention (Augustine and Larsen, 2011; Kahneman and Frederick, 2007; Miu and Crişan, 2011). A few studies, however, have suggested that reappraisal of incidental emotions could be associated with increased risk-taking in a subsequent decision-making task (Heilman *et al.,* 2010; Szasz *et al.,* 2016).

The cognitive control network typically underlying emotion regulation involves lateral prefrontal cortex (dorsolateral and ventrolateral prefrontal cortex, dlPFC and vlPFC), parietal and temporal regions as well as somatosensory cortex (Kohn *et al.,* 2014; Morawetz *et al.*, 2017). Increased responses within this prefrontal network are usually found to be related to decreased responses in the amygdala and striatum (Phillips *et al.,* 2008; Ochsner *et al.,* 2012). Neural networks underlying financial decision-making and preference formation are similarly spread across multiple brain regions, including dlPFC, vlPFC, medial PFC, orbitofrontal cortex, the ventral striatum, anterior cingulate cortex and posterior parietal regions (Bartra *et al.,* 2013; Clithero and Rangel, 2014; Clithero *et al.,* 2009; Kable and Glimcher, 2007; Kuhnen and Knutson, 2005; Levy and Glimcher, 2012; Voigt *et al.*, 2018). The regulation of emotions during monetary incentive tasks has been linked to activity in prefrontal regions, such as the dlPFC and the vmPFC (Sokol-Hessner *et al.*, 2013; Staudinger *et al.*, 2011) and the striatum and the amygdala (Martin and Delgado, 2011; Sokol-Hessner *et al.*, 2013; Staudinger *et al.*, 2011). This suggests that the prefrontal regions might be involved in implementing the reappraisal-related changes in the decision-making circuitry by modulating activity in subcortical areas. Based on these findings, it has been suggested that when emotion regulation is used to change current and expected emotions related to a decision, this engages the same neural circuitry that is regularly found in typical emotion regulation studies where individuals down-regulate their emotions in response to aversive stimuli (Phelps *et al.,* 2014). Some of these regions, however, are also part of the decision-making network, leaving it an open question as to how much of this network is shared between functions.

The primary goal of this study was to investigate the effect of regulation of emotional responses to preceding, incidental stimuli and subsequent choice behavior. This research question, which markedly differs from investigating the regulation of decision-related emotional responses to anticipated decision outcomes, allowed for exploring the impact of emotional spill-over effects on decision-making. In everyday life, we are very likely to constantly encounter emotional stimuli that are not directly related to the next decision. This means, understanding how dealing with such emotions can impact the next, apparently unrelated decision process is of utmost importance.

We used a typical emotion regulation task (e.g. Morawetz *et al.*, 2016a and 2016b), which was followed by a financial risky decision-making task (Mohr *et al.,* 2010; Majer *et al.,* 2016). Participants’ neural responses were measured using functional magnetic resonance imaging (fMRI) as well as participants’ skin conductance to quantify physiological arousal responses to relate those to emotional reactivity and regulation.

Given that the engagement of highly similar neural circuitry is found in typical emotion regulation tasks and tasks involving reappraisal to change current/expected emotions and choices, we hypothesized that carry-over effects of incidental emotions and their regulation during decision-making would be represented in systematic activity changes in shared regions.

## Materials and methods

Here we report two studies: a behavioral experiment to investigate the effect of emotion regulation on decision-making, followed by an fMRI experiment, which served to investigate the neural correlates of this process. In two independent samples, the two experiments used identical experimental paradigms, which are therefore described together in this section.

### Participants

Behavioral experiment: We tested 22 right-handed, healthy participants with normal or corrected to normal vision (18 female, mean age = 22.77 years, SD = 5.28).

fMRI experiment: We tested 33 right-handed, healthy participants with normal or corrected to normal vision. Two participants had to be excluded due to technical problems with data acquisition and two other participants were not able to finish the experiment. The final sample consisted of 29 participants (13 females, mean age = 24.52 years, SD = 4.25).

Participants in both experiments gave written, informed consent to participate. The studies were approved by the local ethics committee of the Department of Education and Psychology at Freie Universität Berlin.

### Stimuli

#### Stimuli for emotion regulation

Stimuli consisted of 96 aversive [normative International Affective Picture System (IAPS) ratings on a Likert scale from 1 (very negative/very calm) to 9 (very positive/very arousing): mean valence = 2.36, mean arousal = 6.30] and 48 neutral (normative IAPS ratings: mean valence = 5.26, mean arousal = 3.37) pictures from the IAPS (Bradley and Lang, 2007). During the experiment, images were presented in the center of the screen with an 800 × 600 pixel display subtending 32° × 24° visual angle on dual display goggles (VisuaStim, MR Research, USA) using the stimulation software Presentation (Version 14.1, Neurobehavioral Systems, USA). Pictures subtended a 24° × 18° visual angle, presented against a black background.

#### Experimental design and procedure

The first part of each experimental trial was a classical emotion regulation task, which has been adapted from previous studies on emotion regulation (Morawetz *et al.,* 2016a, 2016b, 2017). This was followed by a risky decision-making task. During the instruction phase of the experiment, we explained the emotion regulation task and the risky decision-making task in great detail to each participant in written format as well as verbally. Participants performed a short training session before the actual experiment, and they could ask questions if they were uncertain about any aspects of the task.

#### Emotion regulation task

**Fig. 1:**
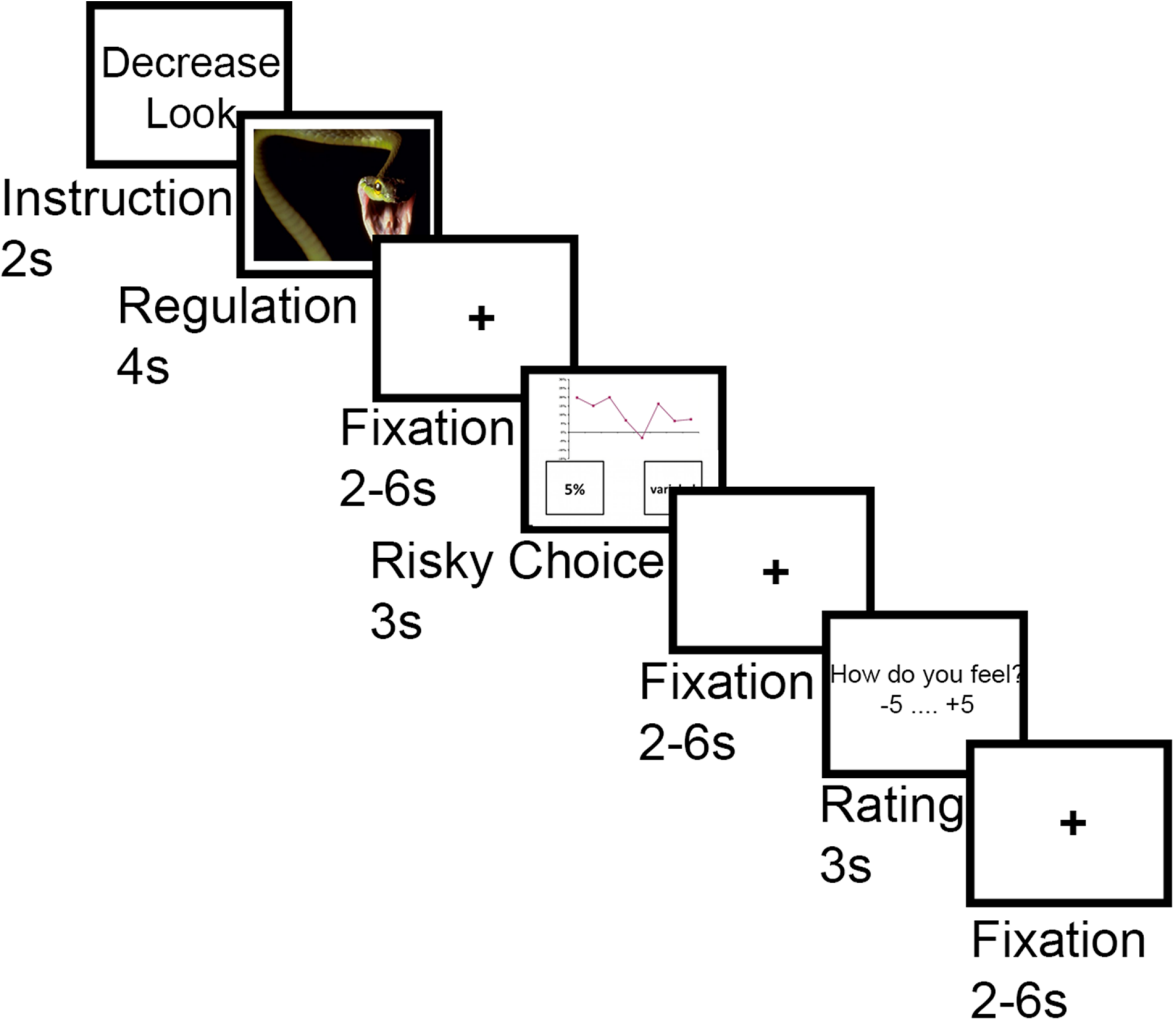
Task design. The first part of each trial was an emotion regulation task, which started with an instruction for 2 s, instructing the participants to either down-regulate (Decrease) their emotional responses or to simply experience them (Look). This was followed by a negative (Decrease and Look) or neutral (Look only) picture presented for 4 s during which the instructed strategy was applied. After a fixation period (2–6 s), the risky decision-making task followed. Participants were presented with a possible investment in a portfolio. The past performance of this portfolio was shown. Participants were asked to choose between the presented portfolio with varying risk (risky option) or a portfolio with a fixed 5% return (safe option, not shown) within a 3 s period. After the decision phase, a fixation period followed (2–6 s). Finally, participants rated their current emotional state (very negative to very positive) within 3 s. The trial concluded with a fixation period (2–6 s).

We used a well-established emotion regulation task, in which participants regulated their emotions in response to viewing one of the pictures in each trial. Three task conditions were implemented (Figure 1): in the Look-Negative condition, participants were presented with aversive pictures and were asked to view the stimuli attentively and allow themselves to experience/feel any emotional responses, which these might elicit without manipulating them. The Look-Neutral condition was identical; however, the stimuli presented did not elicit negative emotions. In contrast, in the Decrease condition, participants viewed negatively valenced images and were asked to actively reduce the intensity of negative emotions by distancing themselves from the image by becoming a detached observer, e.g. through thinking that the depicted situation is not real, by reducing the personal relevance of the image or by telling themselves that the depicted situation is ‘only a picture’ (Ochsner *et al.,* 2004; Eippert *et al.,* 2007; Urry *et al.,* 2009). Importantly, participants were told not to substitute negative emotions with positive emotions.

#### Risky decision-making task

The emotion regulation task was followed by an adjusted version of the Risk Perception in Investment Decisions task (Mohr *et al.,* 2010; Majer *et al.,* 2016). In this task, participants were presented with the course of returns of an investment, i.e. the past performance of a possible investment. The standard deviation of the return streams and the expected returns varied parametrically with four standard deviations (σ = 2%, 4%, 6% and 8%) and four expected returns (μ = 5%, 7%, 9% and 11%), resulting in 16 different combinations of standard deviations and expected returns (see Supplementary Material, Figure S1, for probability distributions). On each trial, participants made a choice between an investment with 5% fixed return (safe investment) and the investment represented by the return stream shown on the screen (risky investment). Participants received a flat payment of 15 euros for their participation in the experiment and a virtual endowment of 100 euros to invest. They were explicitly told that the returns they observe during the experiment were randomly drawn from Gaussian distributions. They were further instructed that after the experiment, one of their 144 choices would be randomly chosen to determine decision-dependent payments. For example, if the participant would choose the safe option in the respective trial, she would receive 5 euros (5% of 100 euros) in addition to the 15 euros flat payment. If a participant would choose the risky option in this trial, a random return was drawn from a Gaussian distribution with the same mean and standard deviation as the respective return stream. The resulting outcome (return times 100 euros) was added to or subtracted from the flat payment. Thus, participants could either win or lose when choosing the risky option. The optimal strategy was therefore to treat each decision as a real decision as each had the same probability to be paid out in the end. Participants could gain a maximum of 21 euros (in addition to the reimbursement for participation) or lose up to 4 euros.

#### Task procedures

In the experiment, both tasks were combined in each trial, i.e. the emotion regulation task was presented first, followed by the risky decision-making task. Each trial started with an instruction cue (2 s) indicating the experimental condition by displaying Decrease or Look (note that Look included both the negative and neutral image condition). Subsequently, an image was presented for 4 s during which the instructed strategy had to be applied. This was followed by a fixation cross for a jittered duration of 2–6 s. After this, participants were presented with the past returns of a new possible investment (risky choice option) on the top of the screen. Underneath, two boxes were displayed on the left or the right side of the fixation cross, indicating the two choice options (safe investment *vs* risky investment). Participants were asked to choose between the safe and risky option by pressing a button on a two-button fiber optic response pad (fORP, Cambridge Research Systems Ltd). The response window was 3 s, and the location of the choice options on the screen was pseudo-randomized between trials to avoid order effects. This task phase was again followed by a jittered fixation period for 2–6 s. Next, subjects were asked to rate their current emotional state on a scale from −5 to +5 (extremely negative to extremely positive) by pressing a button on the two-button fORP, providing a measure of trial-by-trial emotion regulation success. The extremes of the emotional state ratings were not labeled; only the scale from −5 to +5 was presented. The response window for the rating was again 3 s. Finally, a fixation cross was presented in the middle of the screen for a jittered duration of 2–6 s concluded the trial.

Participants performed three runs of the experiment. Each run consisted of 48 trials, and each of the 16 resulting return streams was used in each emotion regulation condition in each run. One trial lasted 24 s on average, one run lasted about 19 min and thus one scanning session consisted of 144 trials, which resulted in ~1 h of scanning.

#### fMRI data acquisition

Whole brain functional and anatomical images were acquired using a 3.0 T Magnetom TrioTim MRI scanner (Siemens, Erlangen, Germany) and a 12-channel head coil. A high-resolution 3D T1-weighted dataset was acquired for each subject (176 sagittal sections, 1 × 1 × 1 mm^3^; 256 × 256 data acquisition matrix). Functional images were acquired using a T2*-weighted, gradient-echo echo planar imaging (EPI) pulse sequence recording 37 sections oriented parallel to the anterior and posterior commissure at an in-plane resolution of 3 × 3 × 3 mm^3^ (interslice gap = 0; TE = 30 ms; TR = 2 s; FA = 90°; FoV = 192 × 192 mm^2^; 64 × 64 data acquisition matrix). For each experimental run 573 whole brain volumes were recorded.

## Data analyses

### Behavioral data

#### Behavioral task performance

We calculated reappraisal success scores based on the affect ratings acquired after each trial. Overall reappraisal success was defined as the mean decrease in reported emotion when applying cognitive reappraisal (Decrease) relative to the mean affect ratings of the control condition (Look-Negative), the latter representing the ‘natural’ emotional response to the stimuli (Morawetz *et al.,* 2016a, 2016b; Wager *et al.,* 2008). Reappraisal success scores for Decrease (Decrease minus Look-Negative) for each participant were calculated and used to analyze how these related to decision-making, i.e. whether emotion regulation *per se* affected choice behavior or whether the effect of emotion regulation on choice behavior depended on regulation success.

As a sanity check, we also analyzed electrodermal activity in each condition. Details can be found in the Supplementary Materials (Figure S2).

## fMRI data

### Preprocessing

Functional imaging data analysis was performed using SPM12 (Wellcome Institute for Cognitive Neurology, London, UK). As interleaved slice acquisition was used, slice time correction was included during the preprocessing of the fMRI data (Sladky *et al.,* 2011). In addition, standard preprocessing involved realignment to the mean image, spatial normalization to the standard EPI template (MNI template, as implemented in SPM8) and spatial smoothing with an 8 mm full-width at half-maximum isotopic Gaussian kernel.

### General linear models

#### We used several general linear models (GLMS) to analyze the data

GLM1. A first GLM was estimated to investigate risky decision-making and identify the emotion regulation network. This model included the following regressors: instruction cue (duration 2 s), emotion regulation conditions (Decrease, Look-Negative, Look-Neutral) (duration 4 s), type of choice (Risky Choices, Safe Choices) (duration 3 s), rating phase (duration 3 s). This model included motion parameters as nuisance covariates. The regressors were convolved with a canonical form of the hemodynamic response. Contrast images of brain activations associated with decision-making (Risky Choices > Safe Choices and Safe Choices > Risky Choices) were calculated for each participant and used in a second-level analysis. Contrast images of brain activations associated with emotion regulation (Decrease > Look-Negative; Decrease > Look-Neutral; Look-Negative > Decrease; Look-Neutral > Decrease) and emotion reactivity (Look-Negative > Look-Neutral; Look-Neutral > Look-Negative) were produced for each participant. *T*-statistics for each voxel were thresholded at *P* < 0.05 corrected for multiple comparisons across whole brain with family wise error (FWE) rate.

GLM2. This model was designed to identify regions whose activity increased during risky decision-making as a function of the emotion regulation condition. For this, the regressors for the choice phase were split into more specific regressors compared to the previous GLMs, specifying the choice outcomes in relation to the preceding emotion regulation condition. It included the following regressors: instruction cue (duration 2 s), emotion regulation conditions (Decrease, Look-Negative, Look-Neutral) (duration 4 s), type of choice (Risky Choices_Decrease_, Risky Choices_Look-Negative_, Risky Choices_Look-Neutral_, Safe Choices_Decrease_, Safe Choices_Look-Negative_, Risky Choices_Look-Neutral_) (3 s) and rating (duration 3 s). This model again included motion parameters as nuisance covariates. The regressors were convolved with a canonical form of the hemodynamic response. Contrast images of brain activations associated with risky decision-making following experiencing negative emotions (Risky Choices_Look-Negative_ > Risky Choices_Look-Neutral_; Risky Choices_Look-Neutral_ > Risky Choices_Look-Negative_) and emotion regulation (Risky Choices_Decrease_ > Risky Choices_Look-Negative;_ Risky Choices_Decrease_ > Risky Choices_Look-Neutral;_ Risky Choices_Look-Negative_> Risky Choices_Decrease;_ Risky Choices_Look-Neutral_ > Risky Choices_Decrease_) were calculated and used in a second-level analysis. *T*-statistics for each voxel were thresholded at *P* < 0.05 corrected for multiple comparisons across whole brain with FWE rate.

## Results

### Behavioral study

#### Emotion regulation task

A significant main effect of task was found (*F*(1,21) = 157.87, *P* < 0.001). *Post-hoc t*-tests revealed significantly more negative emotional state ratings for Look-Negative compared to Look-Neutral (*t*(21) = −14.37, *P* < 0.001, Cohen’s *d* = 4.61) and for Look-Negative compared to Decrease (*t*(21) = −10.61, *P* < 0.001, Cohen’s *d* = 1.24). Decrease also resulted in more negative emotional state ratings than Look-Neutral (*t*(21) = −10.71, *P* < 0.001, Cohen’s *d* = 3.22).

#### Risky decision-making task

A significant main effect of task was observed (*F*(1,21) = 4.21, *P* < 0.05). *Post-hoc t*-tests showed that participants chose the safe option more often after emotion regulation (Decrease) as compared to Look-Negative (*t*(21) = −2.11, *P* < 0.05, Cohen’s *d* = 0.24) and as compared to Look-Neutral (*t*(21) = −3.45, *P* < 0.01, Cohen’s *d* = 0.56). Reaction times during the risky decision-making task did not differ after emotion induction and regulation (all *P* > 0.1, data not shown).

### fMRI study

#### Emotion induction

After the fMRI experiment, participants rated all the images on valence and arousal using a nine-point Likert scale from 1 (very negative/very calm) to 9 (very positive/very arousing). The images a priori selected to be perceived as ‘negative’ were indeed rated as more negative (*t*(25) = 15.10, *P* < 0.001, Cohen’s *d* = 4.48) and more arousing (*t*(25) = 11.36, *P* < 0.001, Cohen’s *d* = 3.05) than the ‘neutral’ images, confirming the normative ratings (Bradley and Lang, 2007). The available skin conductance data provided support for the success of the emotion induction (Figure S2).

#### Emotion regulation task

**Fig. 2:**
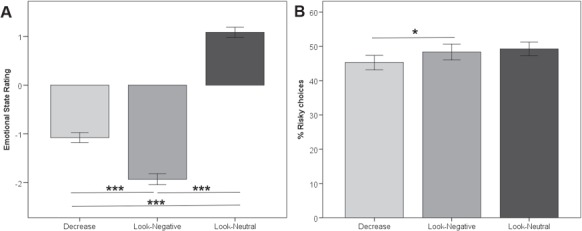
Physiological and behavioral results. (A) Emotional state ratings after each trial (fMRI study). The subjective ratings of emotional states indicated fewer negative emotions after the down-regulation of emotions compared to Look-Negative. During the Look-Neutral condition, participants rated their emotional state to be significantly less negative compared to the Decrease and Look-Negative condition. (B) Percent risky choices as a function of preceding emotion regulation (fMRI study). After emotion regulation, participants showed less risk-taking behavior, i.e. they chose the safe option more often compared to the Look-Negative condition. ^*^ indicates *P* < 0.05; ^**^ indicates *P* < 0.01; ^***^ indicates *P* < 0.001.

A significant main effect of task was found (*F*(1,28) = 103.53, *P* < 0.001) (Figure 2A). *Post-hoc t*-tests revealed significantly more negative emotional state ratings for Look-Negative as compared to Look-Neutral (*t*(28) = −11.00, *P* < 0.001, Cohen’s *d* = 3.48) and for Look-Negative as compared to Decrease (*t*(28) = −7.13, *P* < 0.001, Cohen’s *d* = 1.12). Decrease also resulted in more negative emotional state ratings than Look-Neutral (*t*(28) = −9.75, *P* < 0.001, Cohen’s *d* = 2.76). These results again confirm that emotion regulation was successful.

#### Risky decision-making task

First, we confirmed that participants again preferred the safe over the risky option after Decrease compared to Look-Negative (*t*(28) = −2.34, *P* < 0.05, Cohen’s *d* = 0.16). The differences between Decrease and Look-Neutral (*t*(28) = −1.48, *P* = 0.15, Cohen’s *d* = 0.01) as well as Look-Negative and Look-Neutral (*t*(28) = 0.16, *P* = 0.88, Cohen’s *d* = 0.14) were non-significant (Figure 2B). Reaction times during the risky decision-making task did not differ after Look-Negative and Decrease (all *P* > 0.1; data not shown).

Second, we performed two control analyses to investigate (i) the effect of emotion regulation success on subsequent choices and (ii) the effect of arousal on choices during the decision phase. The results can be found in the Supplementary Materials.

## fMRI results

In a first step (based on GLM1), we performed two control analyses that mainly served as a general sanity check. First, we tested for neuronal correlates of emotion regulation. For this, we performed a conjunction analysis [(Decrease > Look-Negative) & (Decrease > Look-Neutral)]. The results revealed increased activity in the left IFG and the supplementary motor area (SMA) (Table 1). Contrasting the Decrease condition with Look-Neutral revealed increased activity in a widespread network of regions including frontal (IFG), temporal and parietal regions (Table 1). The observed network aligns with our previous findings on the general emotion regulation network (Morawetz *et al.,* 2017). Second, we investigated which regions were implicated in risk-related decision-making in general by contrasting risky *vs* safe decisions (Risky Choices > Safe Choices), independent of the emotion regulation conditions, and found an increase in activity in the vmPFC, the right dlPFC as well as the thalamus (Table 2). These findings are in line with a previous meta-analysis on value-based decision-making (Bartra *et al.,* 2013).

**Table 1:** Emotion regulation task

Contrast	Region	L/R	Size	*t*-value	*P* (FWE-corr.)	Coordinates		
						x	y	z
Conjunction:	SMA	L	58	2.74	0.03	−3	8	62
(Decrease > look-Negative) & (Decrease > Look-Neutral)	Inferior frontal gyrus	L	73	2.63	0.01	−54	26	5
Decrease > Look-Negative	Caudate	R	112	4.89	0.02	9	23	2
	Cerebellum	R	97	4.88	0.04	15	−88	−19
Decrease > Look-Neutral	Inferior occipital gyrus	L	713	7.79	<0.001	−42	−67	−4
	Inferior temporal gyrus	R	321	7.32	<0.001	45	−64	−7
	Supramarginal gyrus	L	182	7.08	<0.001	−63	−31	35
	Precentral gyrus	L	884	5.80	<0.001	−42	−1	35
	Superior parietal lobe	R	317	5.58	<0.001	30	−52	62
	Superior parietal lobe	L	342	5.40	<0.001	−27	−55	59
	SMA	L	180	4.99	<0.001	−3	11	56
	Supramarginal gyrus	R	76	4.92	0.04	63	−25	44
	Precentral gyrus	R	96	4.84	0.01	48	8	38
	Inferior frontal gyrus	R	232	4.82	<0.001	48	26	5
	Thalamus	L	76	4.50	0.04	−6	−10	−1
Look-Negative > Decrease	Middle temporal gyrus	L	857	5.42	<0.001	−57	−37	5
	Insula	R	520	4.73	<0.001	36	−25	17
	Cerebellum	L	115	4.21	0.02	−18	−46	−28
	Inferior frontal gyrus	L	95	4.10	0.04	−45	38	−7
Look-Neutral > Decrease	Superior temporal gyrus	R	782	7.32	<0.001	63	−13	5
	Medial orbitofrontal gyrus	R	1144	6.53	<0.001	3	50	−7
	Superior temporal gyrus	L	870	6.47	<0.001	−57	−10	2
	Precentral gyrus	L	133	5.34	0.005	−24	−25	77
	Superior frontal gyrus	R	86	5.07	0.02	30	29	53
	Cuneus	R	101	5.03	0.01	15	−58	20
	Postcentral gyrus	R	271	4.85	<0.001	15	−34	80
	Cuneus	L	76	4.20	0.04	−15	−58	20
Look-Negative > Look-Neutral	Supramarginal gyrus	L	144	9.08	<0.001	−63	−31	38
	Middle temporal gyrus	L	462	8.78	<0.001	−48	−67	2
	Inferior temporal gyrus	R	210	7.63	<0.001	48	−64	−7
	Vermis		153	7.31	<0.001	−3	−31	−4
	Superior occipital gyrus	R	48	6.78	<0.001	27	−70	32
	Superior parietal lobe	R	102	6.67	<0.001	27	−61	56
	Inferior frontal gyrus	R	32	5.92	<0.001	36	29	2
	Superior occipital gyrus	L	28	5.88	<0.001	−24	−73	32
	Precentral gyrus	L	46	5.83	<0.001	48	8	38
	Precentral gyrus	R	41	5.77	<0.001	48	8	38
	Medial superior frontal gyrus	R	15	5.74	0.001	6	47	44
	Superior parietal lobe	L	63	5.74	<0.001	−24	−61	56
	Supramarginal gyrus	R	15	5.73	0.001	63	−22	38
	Inferior frontal gyrus	R	29	5.44	<0.001	45	23	20
	Insula	R	11	5.30	0.002	42	8	−16
Look-Neutral > Look-Negative	SMA	L	777	6.21	<0.001	−9	−16	56
	Superior temporal gyrus	L	138	5.83	0.004	−54	−10	−1
	Superior temporal gyrus	R	289	5.33	<0.001	60	−13	2
	Insula	L	122	4.63	0.007	−36	−22	23

L = left. R = right. Coordinates refer to MNI coordinate system. *P* < 0.05 FWE corrected (*k* = 10).

**Table 2:** Risky decision-making task

Contrast	Region	L/R	size	*t*-value	*P* (FWE-corr.)	Coordinates		
						x	y	z
Risky Choices > Safe Choices	Anterior cingulum/vmPFC	L	196	5.08	0.001	−3	41	8
	Thalamus	R	105	4.50	0.02	6	−7	5
	Superior frontal gyrus/dlPFC	R	80	4.25	0.05	18	41	44
Safe Choices > Risky Choices	No significant clusters							
Risky Choices_Decrease_ > Risky Choices_Look-Neutral_	Superior frontal gyrus/dlPFC	L	176	4.55	0.003	−24	41	32
	Cingulum	L	109	4.50	0.02	−21	−46	29
	Inferior occipital gyrus	R	94	4.20	0.03	39	−88	−4
	Inferior frontal gyrus/vlPFC	L	117	3.87	0.01	−60	17	17
Risky Choices_Look-Neutral_ > Risky Choices_Decrease_	No significant clusters							
Risky Choices_Decrease_ > Risky Choices_Look-Negative_	No significant clusters							
Risky Choices_Look-Negative_ > Risky Choices_Decrease_	No significant clusters							
Risky Choices_Look-Negative_ > Risky Choices_Look-Neutral_	Superior frontal gyrus/dlPFC	R	116	4.37	0.01	21	59	20
	Superior frontal gyrus/dlPFC	L	82	4.38	0.05	−24	50	29
Risky Choices_Look-Neutral_ > Risky Choices_Look-Negative_	No significant clusters							
Safe Choices_Decrease_ > Safe Choices_Look-Neutral_	No significant clusters							
Safe Choices_Look-Neutral_ > Safe Choices_Decrease_	No significant clusters							
Safe Choices_Decrease_ > Safe Choices_Look-Negative_	No significant clusters							
Safe Choices_Look-Negative_ > Safe Choices_Decrease_	No significant clusters							
Safe Choices_Look-Negative_ > Safe Choices_Look-Neutral_	No significant clusters							
Safe Choices_Look-Neutral_ > Safe Choices_Look-Negative_	No significant clusters							

L = left. R = right. Coordinates refer to MNI coordinate system. *P* < 0.05 FWE corrected (*k* = 10).

**Fig. 3:**
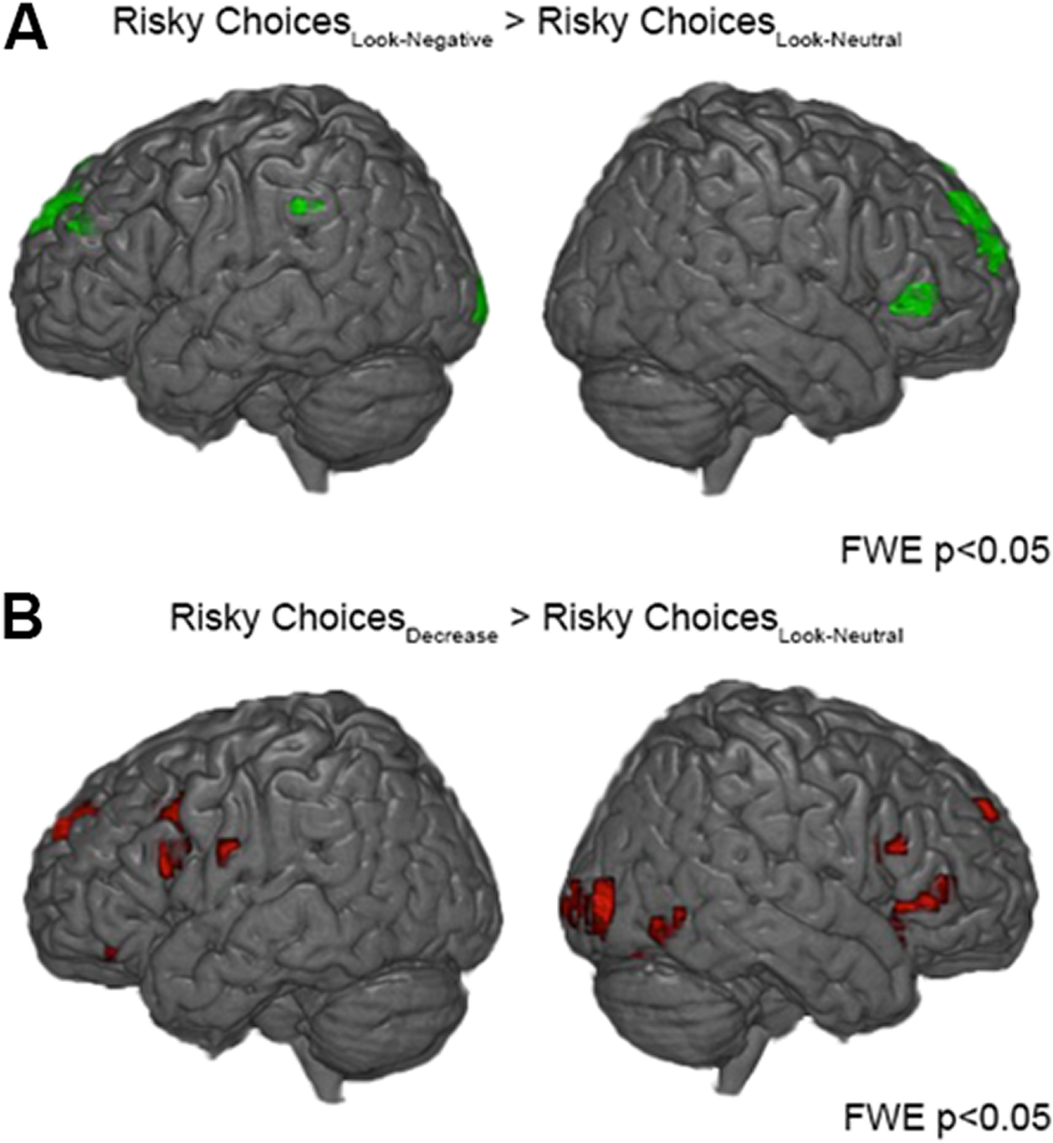
(A) Risky choices after Look-Negative. The contrast (Risky Choices_Look-Negative_ > Risky Choices_Look-Neutral_) yielded increased activity in bilateral dlPFC. (B) Risky choices after emotion regulation. The effect of emotion regulation on risky choices (Risky Choices_Decrease_ > Risky Choices_Look-Neutral_) yielded increased activity in the left dlPFC, left vlPFC and cingulate cortex.

In a second step (based on GLM2), we identified the brain regions linked to risky decision-making when regulating emotions. More specifically, we tested whether the experience of emotional responses affected neural activity during risky decision-making (Risky Choices_Look-Negative_ > Risky Choices_Look-Neutral_). The results revealed increased activity in the bilateral dlPFC (Figure 3A, Table 2). Next, we investigated the neural activity related to the preference for risky options over safe options after emotion regulation. For this, we contrasted (Risky Choices_Decrease_ > Risky Choices_Look-Neutral_) and observed enhanced activity in left dlPFC, left vlPFC and cingulate cortex (Figure 3B, Table 2). The reverse contrasts for neural activity during safe decision-making did not reveal any significant differences in a whole-brain analysis (Table 2).

In a third step, we determined in an explorative manner the overlap of regions implicated in the emotion regulation and risky decision-making in general, by combining the contrasts of both task phases. An overlap in activity between the contrasts (Risky Choices _Decrease_ > Risky Choices_Look-Neutral_) and [(Decrease > Look-Negative) & (Decrease > Look-Neutral)] was found in the left vlPFC region. This suggests that the observed effect of emotion regulation on risky decision-making could be a direct carry-over effect from the emotion regulation task.

## Discussion

Our findings showed that emotion regulation of incidental emotions effectively reduced the experience of negative emotions and subsequently resulted in decreased risk-taking in two independent samples. This carry-over effect of emotion regulation on decision-making was reflected in increased activity in ventrolateral and dorsolateral prefrontal regions and cingulate cortex. Furthermore, we found evidence for a shared neural substrate for emotion regulation and risky decision-making in left vlPFC. Together, these findings suggest that emotion reappraisal can alter risk-related decision-making even if the emotional stimulus is incidental.

## Effect of reappraisal of incidental emotions on risky decision-making

Behaviorally, our results both confirm and contradict previous studies. On the one hand, previous studies support the idea that emotion regulation leads to increased risk-taking behavior (Heilman *et al.,* 2010; Braunstein *et al.*, 2014; Panno *et al.,* 2013), as individuals who successfully regulate their negative emotions tend to make choices that maximize performance (Seo and Barrett, 2007) and place less weight on the outcome of a single decision, which in turn reduces loss aversion (Sokol-Hessner *et al.*, 2013; Sokol-Hessner *et al.,* 2009). On the other hand, Martin and Delgado (2011) reported that reappraisal leads to more goal-directed behavior and promotes less risk-taking behavior. Our findings indicate that reappraisal of current emotions was linked to less risky choices. One pathway for this effect might be that emotion regulation neutralizes the negative emotional state as the decision-maker becomes aware of, and actively manages, their negative affective experience (Forgas, 2000; Forgas and Ciarrochi, 2002). This could then lead to a carry-over effect of enhanced cognitive control into the decision stage and, as a by-product, decrease risk-taking. Note, however, that this interpretation depends on the definition of goal-directedness in this scenario and only holds if resisting the temptation of making a risky choice (as in gambling) is regarded as requiring more cognitive control than the other way around. Alternatively, one might argue that emotion regulation could simply neutralize the impact of the negative emotional experience that would otherwise carry-over into the decision-phase and increase risk-taking. However, our finding that there was no difference in risk-taking between the passive viewing conditions involving negative and neural images clearly contradicts this interpretation. This means, in our study, it was the regulation of a preceding negative experience rather than the negative experience *per se* that impacted subsequent incidental decision-making.

There are three major differences between our study and earlier studies investigating emotion regulation and decision-making that might explain some of the discrepancies in findings. Firstly, we induced incidental negative emotions before each decision, meaning that participants regulated emotional responses that were not related to the decision process *per se*. In contrast, in some previous studies participants were asked to emphasize or deemphasize the importance of an upcoming decision (e.g. Braunstein *et al.*, 2014; Sokol-Hessner *et al.*, 2009; Sokol-Hessner *et al.*, 2013). This means that unlike in our study, in those previous studies no explicit emotional stimulus was present at the time of regulation, rather, the emotions were expected to arise from the decision-making process. Regulating emotions related to potential decision outcomes could arguably lead to a very different profile of choices. Secondly, we instructed participants to use reappraisal to regulate their emotions by implementing tactics such as reality change or distancing (McRae *et al.,* 2012). However, other studies used different strategies to reduce emotions such as taking the perspective of a trader (Sokol-Hessner *et al.,* 2009) or imagine a calming scene such as a sunny day in a park (Martin and Delgado, 2011). These differences in regulation strategy could potentially also lead to differences in choice profiles. Thirdly, we did not provide immediate feedback on participants’ choices that could have triggered emotional processes as in other studies (Sokol-Hessner *et al.*, 2013; Sokol-Hessner *et al.,* 2009; Staudinger *et al.*, 2011; Staudinger *et al.,* 2009). This again fully decoupled the emotional response from the decision-making process and allowed us to isolate incidental regulation-related processes. There is one other study that we are aware of that induced incidental emotions using negative film clips before risky decision-making (Heilman *et al.,* 2010). These authors used the Balloon Analogue Risk Task in which participants can earn financial rewards by ‘pumping up’ balloons presented on the screen. However, as the balloons have variable, unknown explosion points, and participants lose when the breaking point is reached, emotion regulation might also extend to feedback/outcome-related emotions, or expectation of those. In contrast, in our task emotion regulation was more directly attributable to the emotional stimulus alone as no feedback was provided for risky choices.

It is important to note, however, that our study, like most others in the field, did not explicitly investigate the effect of a variety of specific negative (or positive) emotions, or the regulation of such, nor did it systematically vary the type of decision task and risk or include specific groups of decision-makers. It is also conceivable that in other decision scenarios, choosing the risky option might always be the optimal choice, and it is not clear whether the impact of emotion regulation on such choices would be different. It therefore remains to be seen how well our results generalize to other emotion and decision scenarios.

## Neural networks underlying the effect of emotion regulation on risky decision-making

We found that the modulatory effect of cognitive reappraisal of incidental emotions on decision-making was primarily associated with increased activity in dlPFC and vlPFC. Our findings extend previous studies that only reported an increase in activity in the dlPFC when individuals engaged in reappraisal to change emotional responses and choices (Grecucci *et al.*, 2013; Hutcherson *et al.*, 2012; Sokol-Hessner *et al.*, 2013; Staudinger *et al.*, 2011). Moreover, we found that the left vlPFC was implicated in the emotion regulation process as well as in the decision-making process. In the context of emotion regulation, dlPFC has been associated with top-down cognitive control processes involved in attention and working memory (Corbetta and Shulman, 2002; Rottschy *et al.,* 2012; Nee *et al.,* 2013; Vossel *et al.,* 2014; Cieslik *et al.,* 2015), while vlPFC has been implicated in top-down outcome-based language-related appraisal processes (Kohn *et al.,* 2014; Liakakis *et al.,* 2011; Messina *et al.,* 2015; Morawetz *et al.,* 2016a). Both regions seem to be part of a self-regulating feedback loop during reappraisal (Morawetz *et al.,* 2016b).

Our findings suggest that the impact of emotion regulation on the decision process is modulated by this shared neural network. Of course, our results do not provide direct evidence that both processes are neurally integrated in these regions, but the shared cognitive functions, in particular in cognitive control processes, might suggest such an explanation. A pre-activation of cognitive control regions during emotion regulation could lead to spill-over effects to cognitive control in risk-related decision-making, again lending support to the view that it was the regulation process and not the absence of negative emotions that was related to the altered choice patterns. Thus, our findings suggest that emotion regulatory processes in general play a critical role in value computation, and that changes in emotional states can be associated with changes in choice outcomes, even when the emotions are not directly related to any aspects of the decisions *per se*.

## Conclusions

Our results extend earlier work on emotion regulation and decision-making in two ways: first, we showed that emotion regulation of incidental emotions affected risk-taking in decision-making, potentially because cognitive control processes carried over to the decision stage in the absence of immediate outcome feedback. Second, our study provides insights into the possible neural networks underlying emotion regulation of incidental emotions and subsequent decision-making, supporting the view of multiple modulatory neural circuits (Phelps *et al.,* 2014). Our study provides a first step toward a more nuanced understanding of the relationship between emotion regulation and decision-making.

## Funding

This work was supported by the Deutsche Forschungsgemeinschaft Grant MO 2041/2-1 to C.M. and an Australian Research Council Discovery Project grant DP160103353 to S.B.

## Conflict of interest

None declared.

## Supplementary Material

scan-18-407-File007_nsz078
