# Supplementary material for: The effect of emotion regulation on risk-taking and decision-related activity in prefrontal cortex
Source: Soc Cogn Affect Neurosci. 2019 Oct 31;14(10):1109–18. doi: 10.1093/scan/nsz078 (PMC6970147; doi:10.1093/scan/nsz078)
Supplement: scan-18-407-File007_nsz078 [file scan-18-407-file007_nsz078.docx]

**The effect of emotion regulation on risk-taking and decision-related activity in prefrontal cortex**

Carmen Morawetz^1,2,3^, Peter N.C. Mohr^1,4,5^, Hauke R. Heekeren^1,2^, Stefan Bode^6,7^

1 Department of Education and Psychology, Freie Universität Berlin, Germany

2 Center for Cognitive Neuroscience Berlin, Freie Universität Berlin, Germany

3 Center for Medical Physics and Biomedical Engineering, Medical University of Vienna, Austria

4 School of Business & Economics, Freie Universität Berlin, Germany

5 Markets and Choice, WZB Berlin Social Science Center, Germany

6 Melbourne School of Psychological Sciences, The University of Melbourne, Australia

7 Department of Psychology, University of Cologne, Germany

1. **Methods**

**1.1 Details on Risky Decision-Making Task**

In the following section, we provide details on the generation of the investments presented in the risky decision-making task.

Each point of the investment (n-1) was randomly selected from a normal distribution of the denoted expected value and the standard deviation. The last point of the investment was generated by comparing the empirical mean of the rate of return with the theoretical expected value of the distribution. Using this procedure 1000 samples were generated. A sequence of return rates was selected for the experiment in which the empirical standard deviation of the distribution was approximately equal to the theoretical one. This means that the empirical expected value and standard deviation a) approximated in each trial the theoretical ones, b) were predetermined, c) were identical for all participants and d) were not computed ad hoc for each trial. The presentation of the return rates was pseudorandomized across runs.

Please see **Figure S1** below depicting probability density graphs for each of the four returns and respective standard deviations (first row: EV=5, second row: EV=7, third row: EV=9, and fourth row: EV=11). The Figure illustrates the probability of the returns (y-axis) and the possible gains in EUR (x-axis). It is important to note that participants were not informed of the range of the expected returns (-4 to +21). Higher SD (6 and 8) was related to higher gains (20-25 EUR), while lower SD (2 and 4) was associated with medium (8-15 EUR) and low gains (5-6 EUR). At the same time, expected returns with a high SD of 8 also included the highest probability of losing money.


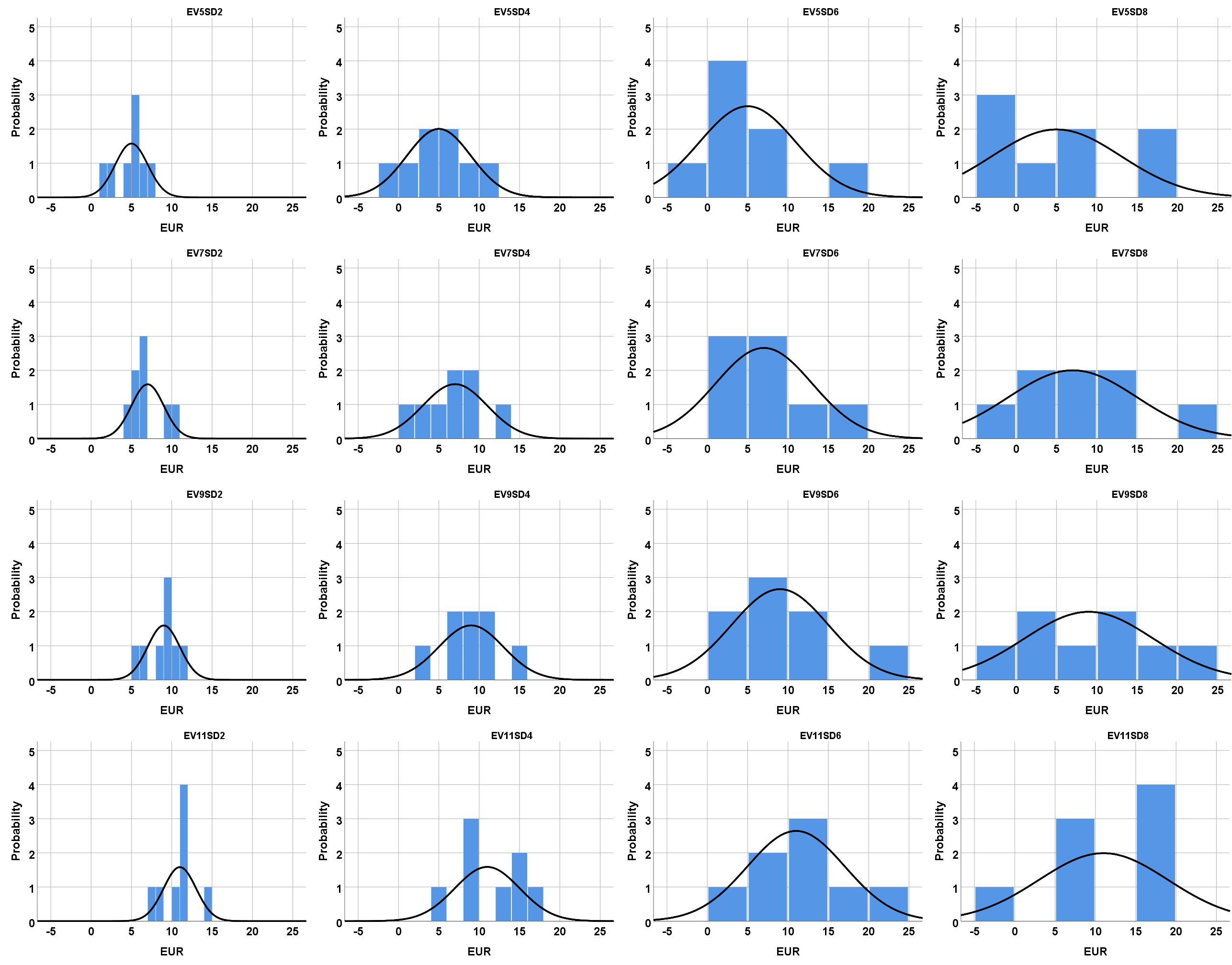


**Figure S1.** Probability of returns. Rows correspond expected returns (first row: EV=5, second row: EV=7, third row: EV=9, and fourth row: EV=11) and columns to standard deviations (first column: SD=2, second column: SD=4, third column: SD=6, fourth column: SD=8).

**1.2 EDA analysis**

The data were down-sampled offline to 10Hz and averaged across trials within each condition applying an 8s time window using Ledalab Version 3.3.1 (Benedek and Kaernbach, 2010a). Continuous decomposition analysis was performed to decompose skin-conductance data into continuous tonic and phasic activity (Benedek and Kaernbach, 2010b). Skin-conductance responses (SCRs) were defined as a deflection of at least 0.01 µS occurring 1-4s after stimulus onset. Only runs including more than 10% SCRs exceeding the above criterion were used for analysis. Values for phasic SCRs were extracted as the difference between a local minimum and the succeeding local maximum within the response window. EDA data from nine participants had to be excluded due to technical problems at recording (total sample size of n=19). All runs from the final sample were included in the analysis.

1. **Results**
   1. **Skin conductance**

Skin conductance data provided support for the success of the emotion induction during the image presentation phase (**Figure S2**). Note, due to technical problems at recording, data from nine participants could not be included in the analysis (total sample size of n=19). A repeated measures ANOVA (task: Decrease, Look-Negative, Look-Neutral) revealed a significant main effect of task (F(1,18)=5.08, p<0.01). Post-hoc t-tests indicated that SCRs during Look-Negative were higher compared to Look-Neutral (t(18)=-2.41, p<0.05, Cohen’s d=0.37). Moreover, the SCR amplitudes were significantly higher during Look-Negative compared to Decrease (t(18)=2.21, p<0.05, Cohen’s d=0.16), in accord with previous findings (Urry et al., 2009). Note that due to technical problems at recording, the final small sample size for this analysis was low (n = 19), and these findings have to be interpreted with caution.


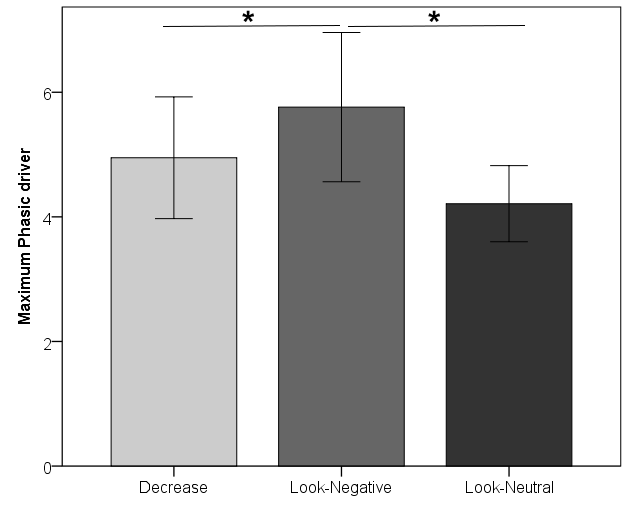


**Figure S2.** Skin conductance responses during the emotion regulation task as a function of regulation condition. Skin conductance responses were attenuated by reappraisal compared to the Look-Negative condition. During Look-Negative skin conductance responses were higher compared to Look-Neutral. * indicates p<0.05; ** indicates p<0.01; *** indicates p<0.001.

- 1. **Control analyses**

1. To test whether choice behaviour was affected by emotion regulation success before the decision phase, we performed a repeated measures ANOVA with the factors ‘regulation success’ (successful, unsuccessful) and ‘choice’ (risky, safe). For this, we calculated the emotion regulation success during the Decrease condition on a trial-by-trial basis by subtracting each rating of the Decrease trials from the mean of all Look-Negative trials. This means, a trial was considered successful if the rating during the Decrease condition was less negative than during the mean of the Look-Negative condition. The analysis revealed no significant interaction effect between regulation success and choice (F,(1,28)=0.44, p=.51).

However, it is important to note that regulation success was high on average. This means that regulation success cannot be fully dissociated from the process of emotion regulation per se, and these results have to be interpreted with caution. It remains possible that high regulation success might have been the by- (or end-) product of trials in which the emotion regulation process optimally unfolded, and the effect on choice is therefore still attributable to the cognitive process rather than to its effect. Future studies are needed to address these issues more directly.

1. To test for differences in arousal during the choice period indexed by skin conductance responses, we performed repeated measures ANOVA with the factors regulation (Decrease, Look-Negative, Look-Neutral) and choice (safe, risky) on the EDA data. There was no significant interaction effect between regulation x choice, indicating that emotions and their regulation had no sustained effect on skin conductance responses carrying over into the choice period (F(1,18)=1.51, p=0.234).

**References**

Benedek, M., Kaernbach, C., 2010a. A continuous measure of phasic electrodermal activity. J. Neurosci. Methods 190, 80–91.

Benedek, M., Kaernbach, C., 2010b. Decomposition of skin conductance data by means of nonnegative deconvolution. Psychophysiology 47, 647–658.

Urry, H.L., van Reekum, C.M., Johnstone, T., Davidson, R.J., 2009. Individual differences in some (but not all) medial prefrontal regions reflect cognitive demand while regulating unpleasant emotion. Neuroimage 47, 852–63. https://doi.org/10.1016/j.neuroimage.2009.05.069
